# Tiny but Fatty: Lipids and Fatty Acids in the Daubed Shanny (*Leptoclinus maculatus*), a Small Fish in Svalbard Waters

**DOI:** 10.3390/biom10030368

**Published:** 2020-02-28

**Authors:** Svetlana A. Murzina, Svetlana N. Pekkoeva, Ekaterina A. Kondakova, Zinaida A. Nefedova, Kseniia A. Filippova, Nina N. Nemova, Alexei M. Orlov, Jorgen Berge, Stig Falk-Petersen

**Affiliations:** 1 Environmental biochemistry lab, Institute of Biology of the Karelian Research Centre of the Russian Academy of Sciences, Pushkinskaya st., 11, 185910 Petrozavodsk, Russia; pek-svetlana@mail.ru (S.N.P.); znefed@krc.karelia.ru (Z.A.N.); bystrovakseniia@gmail.com (K.A.F.); nnnemova@gmail.com (N.N.N.); 2 Biological faculty of the St. Petersburg State University, University Embankment 7/9, 199034 St. Petersburg, Russia; katekondakova1989@gmail.com; 3 Federal State Scientific Establishment “Berg State Research Institute on Lake and River Fisheries” (GosNIORH), St. Petersburg branch of VNIRO, Russian Federal Research Institute of Fisheries and Oceanography, Makarova Embankment 26, 199053 St. Petersburg, Russia; 4 Russian Federal Research Institute of Fisheries and Oceanography, Verhnyaya Krasnosel’skaya, 17, 107140 Moscow, Russia; orlov@vniro.ru; 5 A.N. Severtsov Institute of Ecology and Evolution, Russian Academy of Sciences, 33, Leninsky Prospekt, 119071 Moscow, Russia; 6 Department of ichthyology and hydrobiology, Biological Institute, Tomsk State University, 36, Lenina St., 634050 Tomsk, Russia; 7 Faculty of Biosciences, Fisheries and Economics, University of Tromsø, N-9037 Tromsø, Norway; jorgen.berge@uit.no; 8 Akvaplan-niva, Fram Centre, Hjalmar Johansensgt. 14, NO-9296 Tromsø, Norway; stig.falk.petersen@akvaplan.niva.no

**Keywords:** lipids, fatty acids, *Leptoclinus maculatus*, ontogenesis, adaptations, trophic nets, Arctic

## Abstract

The seasonal dynamic of lipids and their fatty acid constituents in the lipid sac and muscles of pelagic postlarval *Leptoclinus maculatus*, an ecologically important fish species in the Arctic food nets, in Kongsfjord, Svalbard waters was studied. The determination of the qualitative and quantitative content of the total lipids (TLs), total phospholipids (PLs), triacylglycerols (TAGs), cholesterol (Chol), cholesterol esters (Chol esters) and wax esters was analyzed by TLC, the phosphatidylserine (PS), phosphatidylethanolamine (PE), phosphatidylinositol (PI), phosphatidylcholine (PC), lysophosphatidylcholine (LPC) and sphingomyelin (SM) were determined by HPLC, and fatty acids of total lipids using GC. The lipid sac is a system of cavities filled with lipids, and it is not directly connected to organs of the digestive system. The wall’s inner layer is a multinuclear symplast that has a trophic function. The results provide additional knowledge on the role of lipids in the biochemical and physiological adaptation of fish to specific environments and clarify the relationship between fatty acids and the food specialization of postlarvae. Analysis of the fatty acid (FA) profile of TLs in the muscles and lipid sac of daubed shanny pelagic postlarvae showed it to be tissue- and organ-specific, and tightly associated with seasonal variations of environmental factors (temperature conditions and trophic resources).

## 1. Introduction

Lipids are one of the diverse, multifunctional, and basic groups of biochemical molecules. Among the numerous functions of lipids in the body, we can distinguish the following primary functions: in any cell, lipids are the basis of biological membranes, where proteins also act (lipids as structural molecules); lipids provide energy for metabolic reactions and processes (acting as energy producers or providing a storage function); and lipids provide a regulatory function that is carried out by biologically active molecules (eicosanoids). The diverse functions of lipids are carried out both intra- and intercellularly [1,2,3,4,5,6]. Individual lipid classes in the body, particularly those of fish, perform several functions, each of which is crucial in specific ecological and physiological conditions [7]. An organism’s stability and sensitivity to various external influences or fluctuations of environmental factors is largely determined by peculiarities of lipid metabolism.

Fatty acids (FAs), which are mainly in a bound state in the body, are the most mobile components of lipid molecules and are characterized by multifunctionality [8]. Fatty acid constituents of lipids are incorporated into adaptive reactions relatively quickly, and under both normal and stress-induced conditions, various FAs provide the body with the choice of alternative ways (mechanisms) of response: regulation of the physical–chemical state of biomembranes, changes in enzyme activity without changing protein concentration, and the synthesis of biologically active mediators [9]. A major proportion of the fish lipid FA complexes are long-chain and highly unsaturated FAs, which emphasizes their important functional role in the body [8]. Moreover, Arctic marine organisms, including fish, accumulate high amounts of monounsaturated long-chain FAs (such as C20 and C22) that, together with polyunsaturated FAs (PUFAs), have significant biological effects on organisms, including humans.

Research on the composition and role of lipids and their FA constituents in fish inhabiting northern seas, as well as the processes of lipid transformation and transfer along the food chains of aquatic ecosystems, is essential for understanding the optimal functioning of all metabolic systems of the body in a changing environment (for example, daily, seasonal, and interannual). With the accumulation of lipids, and their dynamics and expenditure peculiarities for northern latitudes, aquatic organisms ensure the maintenance of vital activity and determine an individual survival rate under changing environmental factors and their combination, taking into account the life cycle of an organism.

Despite the severe environment in the Arctic, ichthyofauna is characterized by high plasticity and variability in life strategies and, in general, Arctic boreal marine ecosystems are very productive. The Arctic ichthyofauna is poorly studied in comparison to that of the Antarctic. There is a lack of up-to-date data on the study of lipids in ecological and biochemical adaptations of marine organisms, especially fish in the Arctic, taking into account that Arctic marine ecosystems are lipid-dependent, and lipids are considered vital molecules in northern latitudes.

The Stichaeidae family is an evolutionarily relatively young, extensive, and extremely diverse taxon [10,11,12] characterized by high adaptive capacities as well as plasticity to environmental factors and their fluctuations. The main directions of the research on representatives of this family around the world are aimed at obtaining new data in the fields of taxonomy, general biology, physiology, and biochemistry, studying the processes by which high-latitude aquatic organisms adapt to their environment [13,14]. Fish of the Stichaeidae family dwell in the bottom of the continental shelf in the coastal waters of marine ecosystems. The depth of their habitat varies greatly, and some species live in tidal currents. Thus, fish belong to the eurigaline group. Specific habitat conditions and a wide range of variations in environmental factors for fish from this family determine adaptability and plasticity.

Prolonged spatial continental isolation has led to the formation of suborder endemic species, including the *Lumpenus* and *Leptoclinus* genera that, among other things, define the northwestern Atlantic region of this suborder endemism [12,15,16]. The daubed shanny *Leptoclinus maculatus* (Fries, 1838) is a marine demersal species belonging to the Arctic boreal [17,18,19] zoogeographical group. Information about the biology of these species from the Stichaeidae family is not abundant [20,21,22,23,24,25,26]. Earlier [27,28,29], it was found that pelagic daubed shanny juveniles have a unique formation, namely, a lipid sac in the ventral part of their body. The lipid sac is a depot of lipids that ensures buoyancy and, to some extent, phenotypic adaptation to pelagic conditions. It consists of large, densely packed lipid drops [27,28,30]. Later [28,29], it was found that postlarvae inhabit the pelagic zone until the age of 3–5 years, after which juveniles switch to a demersal mode of life. Several postlarval stages (L1–L5) have been distinguished for *L. maculatus* on the basis of age, size and weight, body color, and pigmentation, as well as the presence and parameters of the so-called lipid sac [28,29]. The lipid sac is resorbed after the fish become demersal, whereas in the pelagic young, it is distinct and formed by accumulating lipids from a diet of zooplankton (*Calanus*), which is their principal source of food [27,29,31,32,33].

The ecological role of *L. maculatus* becomes clearer upon a detailed examination of the trophic structure of Arctic and boreal waters, in which these species are among the main food sources for marine fish, mammals, and birds [34,35,36]. Studied fish species occupy a double niche in Arctic trophic chains, acting as both predator and prey [37]. The only detailed study of the contribution of the Stichaeidae family, including *L. maculatus* and *Lumpenus fabricii*, to the nutrition of northern birds [38] are results of the study of behavioral reactions of *Cepphus columba*, or pigeon guillemot, when feeding chicks in the Alaskan region, and includes an analysis of the composition and selection of objects as food. Despite the fact that some birds specialize in catching lipid-rich fish (for example, the northern Pacific sand lance *Ammodytes hexapterus* and Pacific herring *Clupea pallasii*), most prefer to produce food for chicks with fish species that are available and constant in the coastal zone, despite their lower calorie content, such as adults of Stichaeidae family.

The aim of the present study was to study the seasonal dynamics of lipids and fatty acids in the lipid sac and muscles of pelagic postlarval *L. maculatus* in Kongsfjord, Svalbard waters. The results can clarify the role of lipids in the adaptation of young fish to specific environments, and the relationship between fatty acids and postlarval food specialization.

## 2. Material and Methods

### 2.1. Sample Collection and Description

*Leptoclinus maculatus* pelagic postlarvae were collected in summer, winter, and spring in Kongsfjord (79°01’, 11°21’), Svalbard, using pelagic and bottom trawls on board the R/V *Helmer Hanssen* (UiT, Norway). The hydrological characteristics of the sampling sites are presented in Table 1.

**Figure 1:**
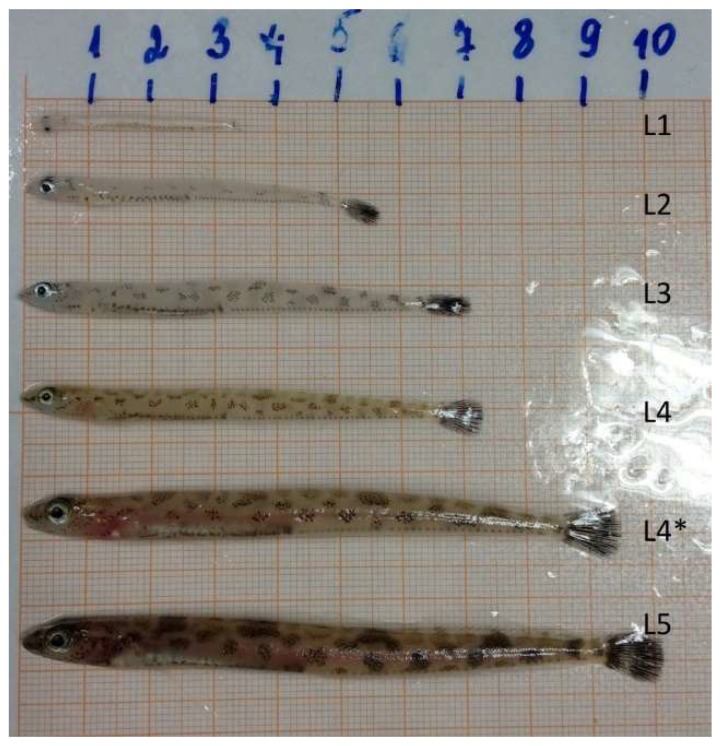
Stages of development of *L. maculatus* by Pekkoeva S.N., 2018 [29].

Daubed shanny juveniles are characterized by long-term development in the pelagic zone and relatively recently established multistage; stages are indicated by a Latin letter and number in the proposed system, and range from L1 to L5 [28,39] (Figure 1). Stages L1–L3 were mainly collected by pelagic trawl, and L4–L4 * by bottom trawl.

Postlarval stages of the daubed shanny differ in their morphological and physiological characteristics (length, weight, body color, pigmentation, and the presence and state of the lipid sac), and are also divided according to belonging to ecological groups (pelagic, transitional, demersal) [29,32,39] (Figure 1). The data on postembryonic growth and the developmental features of the juvenile *L. maculatus* and, in particular, changes in the length and state of the lipid sac during larval development, were presented for the first time in the work of S. N. Pekkoeva [29]. These data are unique for this species as they largely clarify some features of its ecology.

**Figure 2:**
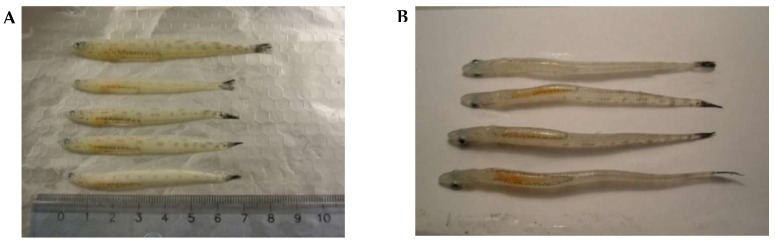
Pelagic postlarvae of *L. maculatus* (L3 stage of development) in summer. (**A**) A side view, (**B**) A view down the lipid sac.

In the present research, lipid profiling was performed on postlarvae in the L3 developmental stage (Figure 2).

Histomorphological studies of the lipid sac were presented on postlarvae in L2–L5 developmental stages.

All work was performed according to and within the regulations enforced by the Norwegian Animal welfare authorities and no specific permissions were required. The R/V Helmer Hanssen is owned by the University of Tromsø and has all necessary authorization from the Norwegian Fisheries Directorate to use a bottom trawl to collect fish for scientific purposes. Furthermore, the organisms are neither protected nor endangered in the coastal waters of the Svalbard Archipelago. Upon trawling, the fish were sacrificed by a sharp blow to the head and immediately dissected as specified below.

### 2.2. Lipid Extraction

The lipid sac and muscle of postlarval *L. maculatus* (L3 developmental stage) were immediately dissected from fresh fish aboard the vessel. Samples were homogenized in glass vials in a chloroform/methanol (2:1, v/v) solution (10 mL per 1–3 g wet weight). Total lipids (TLs) were extracted using the Folch method [40]. The homogenate was filtered, and the residue, retained on the paper filter, was rinsed with 30 mL of extractive mixture. The extract was then mixed after the addition chloroform and deionized water, and left to settle in the separatory glass funnel (Schott Duran, Hamburg, Germany) until complete separation of organic phases. Lipids remained in the lower chloroform layer, whereas non-lipid substances moved to the upper aqueous methanol phase. The chloroform layer was then withdrawn to be evaporated under vacuum on rotary evaporator Hei-VAP Advantage HL/G3 (Heidolph, Schwabach, Germany), and dried in a vacuum exicator over phosphoric anhydride to constant weight. Total lipids were dissolved in chloroform/methanol and stored at −80 °C.

### 2.3. Lipid Class Analysis

Thin-layer chromatography (TLC) was used to identify the lipid classes as total phospholipids (PLs), triacylglycerols (TAGs), cholesterol (Chol), cholesterol esters (Chol esters), and wax esters. Fractionation of total lipids was performed on ultrapure glass HPTLC Silica gel 60 F_254_ Premium Purity plates (Merck, Darmstadt, Germany). Certain lipids, namely, PLs, TAGs, Chol esters, and wax esters, were quantified using the hydroxamate method that was modified by [41], which involves the formation of dark brown complexes of trivalent iron ions with hydroxamic acid through ester bonding between lipids and hydroxylamine [42]. Stain intensity was measured using a spectrophotometer (SF-2000, OKB “Spectr”, St. Petersburg, Russia) at a wavelength of 540 nm. The quantitative determination of Chol was made on the basis of the method described in [43] by using trichloroacetic iron dissolved in perchloric acid. Stain intensity was measured using a spectrophotometer at a wavelength of 550 nm. Standard references and analytical standards for TLC (Sigma Aldrich, St. Louis, MO, USA; Avanti Polar Lipids Co., Alabaster, AL, USA) were used to distinguish the lipid classes.

The spectra of individual phospholipid fractions were determined by high-performance liquid chromatography (HPLC) using Aquilon Stayer HPLC (Aquilon LLC, Moscow, Russia) according to the method of Arduini [44], using a Nucleosil 100-7 C18 HPLC column with a acetonitrile/hexane/methanol/phosphorus acid (918:30:30:17.5, by volume) mobile phase, the rate of movement was 540 mkl/h, the volume of the injected sample was 5 µL, and the injector was Rheodyne 7725i. Detection was performed using a spectrophotometer (UV light, 206 nm), and the method was isocratic. Samples were manually injected using Rheodyne Valco Beckman and SSI Valves syringes (Hamilton, Reno, NV, USA). Phospholipid standards (Sigma Aldrich, St. Louis, MO, USA) were used for the identification and quantification of the phospholipid compounds in the sample. We identified six phospholipids: phosphatidylserine (PS), phosphatidylethanolamine (PE), phosphatidylinositol (PI), phosphatidylcholine (PC), lysophosphatidylcholine (LPC), and sphingomyelin (SM).

### 2.4. Fatty Acid Analysis

The fatty acid profile of the total lipid extracts was analyzed by gas chromatography (GC). The methylation of fatty acids from the lipid extract was made in a glass retort in which 0.1 mL of a solution containing 20 mg/10 mL (behenic FA, C22:0) (Sigma Aldrich, St. Louis, MO, USA) in methanol was added as internal standard; then, we carried out transesterification in methanol (2 mL) containing chlorate acetyl (0.2 mL) at 70 °C for 90 min (using a Schott Duran glass serpentine condenser). After extraction, cooling with hexane was carried out in glass serpentine condensers that were rinsed with 5 mL of hexane for each sample. Then, 2 mL of deionized water was added to each glass retort for phase separation in the separatory glass funnels for 15 min. Fatty acid methyl esters (FAMEs) remained in the upper hexane layer, whereas other substances moved to the lower aqueous phase. The hexane layer was then withdrawn to be evaporated under vacuum on rotary evaporator Hei-VAP Advantage HL/G3 (Heidolph, Schwabach, Germany). Then, 0.9 mL of hexane for GC (Sigma Aldrich, St. Louis, MO, USA) was added to each glass retort, and the content was moved to the glass GC vials for the following GC analysis.

FAMEs were identified using a Chromatek-Crystall-5000.2 (Chromatek, Yoshkar-Ola, Russia) gas chromatograph with a flame-ionization detector (FID) and a Zebron ZB-FFAP capillary gas chromatographic column (Phenomenex, Torrance, CA, USA). An isothermal column configuration was used (200 °C); the temperatures of the detector and evaporator were 250 and 240 °C, respectively. Chromatek-Analytik-5000.2 software (Chromatek, Yoshkar-Ola, Russia) was used for recording and integrating the data. FAMEs were identified with standards of Supelco 37 Component FAME mix, bacterial acid methyl ester (BAME), and PUFA No. 1 (Sigma Aldrich, St. Louis, MO, USA), and by comparing the equivalent lengths of carbon chains and table constants according to Jamieson [45].

### 2.5. Statistical Analysis (Lipid Study)

Data were analyzed to determine whether they exhibited normal distribution. Significant differences (*p* ≤ 0.05) in the mean of the studied lipids and fatty acids between fish collected in different seasons were tested by one-way ANOVA. To perform statistical analysis, StatGraphics (Statpoints technologies, Inc., The Plains, VA, USA) and Microsoft Excel 10 (Windows 7, USA) were used.

### 2.6. Histological Analysis

The lipid sac of postlarval *L. maculatus* (L2–L5 stages) was dissected from fresh material and fixed in 4% formaldehyde (buffered) onboard the vessel. The tails were cut off from postlarval *L. maculatus* (L2 and L3 stages). In the postlarvae at the L4, L4 *, and L5 stages, the heads, pectoral fins, the dorsal part of the body, and tails were removed. In one case, the lipid sac was dissected from the body cavity, but it was found to be dissatisfactory. The material was washed in PBS, dehydrated, and embedded in paraplast (Leica, Wetzlar, Germany) according to the standard procedure. Serial parasagittal sections of 6–7 µm were cut using the sleigh microtome Leica SM 2010R (Leica Microsystems, Wetzlar, Germany). The sections were stained with Carazzi’s hematoxylin and eosin (Biovitrum, St. Petersburg, Russia). Histological sections were studied using the light microscope Leica DMI6000 (Leica, Wetzlar, Germany). Measurements were made using Fiji software [46]. To minimize the risk of remeasuring the same nucleus, at least four sections were left between the measured ones.

### 2.7. Statistical Analysis (Histological Analysis)

Differences in the LSN lengths between L2 and L5 were evaluated using the Mann–Whitney U-test, with *p* < 0.05 indicating significant difference. All data are presented as mean ± SE.

## 3. Results

### 3.1. Histomorphology of the Lipid Sac of Postlarval L. Maculatus (L2–L5 Developmental Stages)

**Figure 3:**
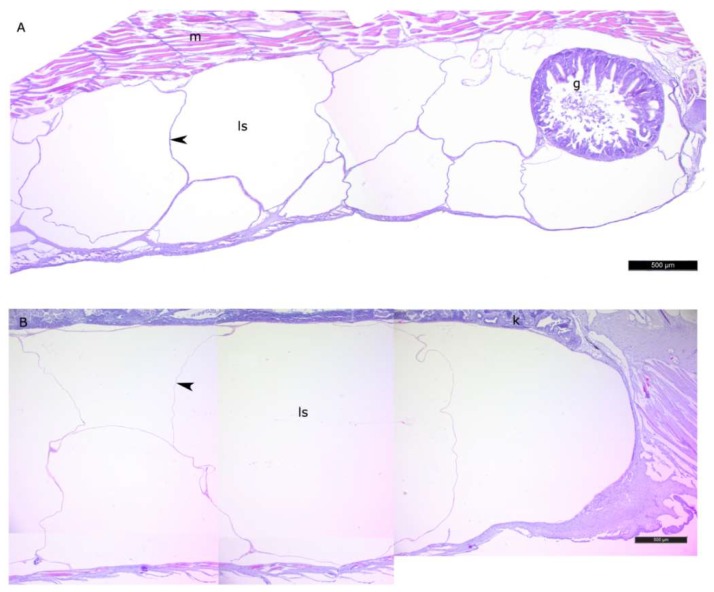
The general view of the lipid sac of postlarval daubed shanny. Parasagittal sections. (**A**)—L2 stage, (**B**)—L4 * stage. Figure legend: g—gut, ls—lipid sac, m—myomere, k—kidney. The walls of the lipid sac cavity are indicated with arrowheads. Staining: Carazzi’s hematoxylin and eosin. Bar: 500 µm.

**Figure 4:**
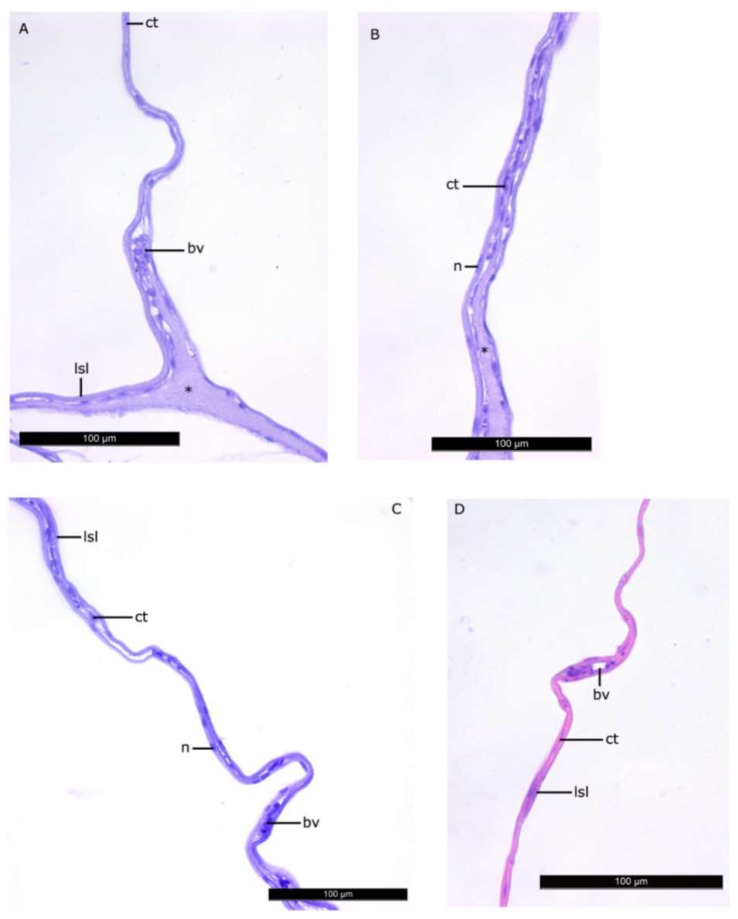
The walls of the lipid sac compartments. Parasagittal sections. (**A**–**C**)—L2 stage, (**D**)—L4 * stage. Figure legend: bv—blood vessel, lsl—lipid syncytial layer, ct—connective tissue, n—nucleus of LSL. The homogenous matrix between the compartments of LS. Staining: Carazzi’s hematoxylin and eosin. Bar: 100 µm.

**Figure 5:**
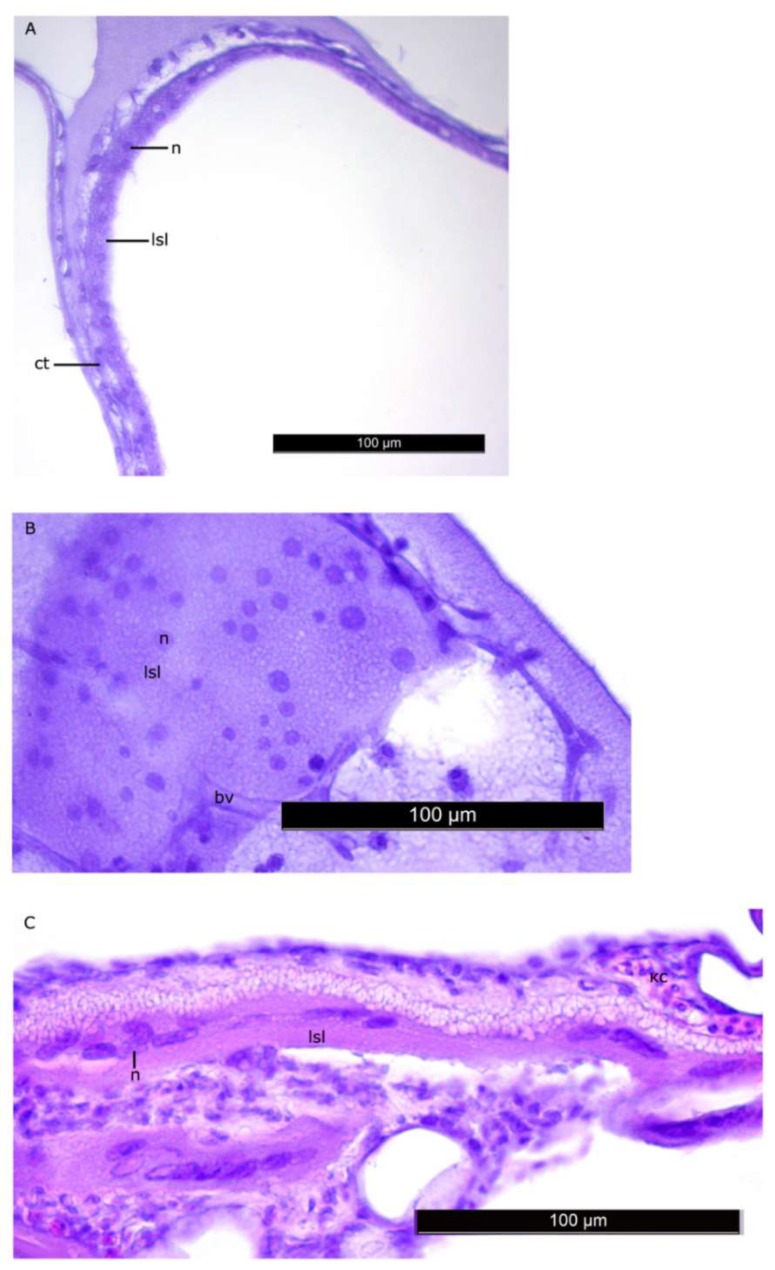
Nuclei of the LSL of LS at the L2 stage (**A**,**B**) and L5 stage (**C**). Nuclei generally have regular shapes, their linear sizes increase by the stage L5. Figure legend: bv—blood vessel, lsl– lipid syncytial layer, ct—connective tissue, n—nucleus of LSL. The homogenous matrix between the compartments of LS. Staining: Carazzi’s hematoxylin and eosin. Bar: 100 µm.

The histomorphological study of the lipid sac (LS) of postlarval *L. maculatus* confirmed that the LS is a complex of lipid-filled compartments that is not directly connected to the digestive system (Figure 3, Figure 4 and Figure 5). The inner layer of these compartment walls is a multinucleate symplast, and the outer layer is a connective tissue [27]. Several layers of connective tissue and blood vessels are found in the walls. We named the LS symplast the lipid syncytial layer (LSL) by analogy with the yolk syncytial layer (YSL). Unstained lipid inclusions were seen in the LSL cytoplasm. The LSL nuclei (LSN) were generally round, elliptical, or elongated in sections. The LSN could be stained weakly or intensively with hematoxylin as a likely indication of differing eu- and heterochromatin content. The linear sizes of the LSN increase significantly by stage L5 (average length of LSN rose from 8.12 ± 0.17 µm at L2 (*n* = 185 where n is the number of nuclei) to 15.94 ± 0.3 at L5 (*n* = 250)).

### 3.2. Seasonal Dynamics of Total Lipids in the Lipid Sac and Muscles of Pelagic Postlarval (L3 Developmental Stage) L. Maculatus

The seasonal variation of total lipid (TL) content in the muscles and lipid sac of pelagic postlarval daubed shanny from Kongsfjord was determined in spring, summer and winter.

TL level was the lowest in muscles in the winter and spring (13.9% and 13.4% dry weight, respectively; no significant differences), whereas the content in muscles in the summer was significantly higher—27.9% dry weight. A different pattern of dynamic in TLs was observed in the lipid sac: the content increased significantly from summer to winter—64.4% and 75.9%, and decreased to 46.96% dry weight by spring (Table 2).

### 3.3. Seasonal Dynamics of Certain Lipid Classes in Lipid Sac and Muscles of Pelagic Postlarval L. Maculatus

The content of storage lipids in the form of triacylglycerols (TAGs), cholesterol esters (Chol esters) and wax esters, and membrane lipids in the form of phospholipids (PLs) in the muscles of the daubed shanny decreased significantly by winter compared to its levels in summer (2.4%, 0.9%, and 7.2% dry weight versus 12.7%, 1.7%, and 9.6%, respectively) (Table 2). The level of TAGs in the fish increased significantly in spring compared to winter (6.1% vs. 2.4% dry weight) but remained lower than in the summer (12.7% dry weight). The level of Chol esters and wax esters in the fish in spring rose to levels observed in the muscles in the summer (1.7% and 1.7% dry weight). The PL content in postlarval muscles was the lowest in spring (2.5% dry weight).

The level of total PLs in *L. maculatus* muscles was associated with variations in both the dominant phosphatidylcholine (PC) and phosphatidylethanolamine (PE) (the former tended to decline while the latter increased from summer towards winter), as well as minor phosphatidylinositol (PI), phosphatidylserine (PS), and sphingomyelin (SM), which arose in the fish from winter towards spring (Table 2). In the muscles of the postlarval daubed shanny, the seasonal variation of total PLs consisted of slight but statistically significant variations of PC and PE, as well as minor PI, PS, and SM, especially in the winter season (Table 2).

The lipid sac of daubed shanny postlarvae had the following changes from summer towards winter: storage TAGs increased from 58.3% to 68.8% dry weight, and trace amounts of Chol esters and wax esters increased from 0.1% to 3.0%, remaining at this level towards spring (2.6% dry weight). Total PL levels declined towards winter (from 6.1% in summer to 0.8% in winter), and by spring, PL content reached a level observed in the young in summer (6.3% vs. 6.1% dry weight). The Chol content in the lipid sac of postlarvae tended to rise from summer towards spring from trace amounts (0.1%) in summer to 3.4% in winter and 10.0% dry weight in spring. The Chol/PL ratio was the highest in the lipid sac of postlarvae in the winter season (4.1), the lowest in summer (0.01), and intermediate in spring (1.6) (Table 2). In spring, when the trend was a general decrease in TLs and TAGs in the lipid sac, the Chol/PL ratio (1.6) could be considered as an indicator of lipid components being utilized for maintaining the viability of postlarval fish (and, hence, the extraction of lipids from the sac).

### 3.4. Seasonal Dynamics of Certain Fatty Acids in the Lipid Sac and Muscles of Pelagic Postlarval L. Maculatus

**Figure 6:**
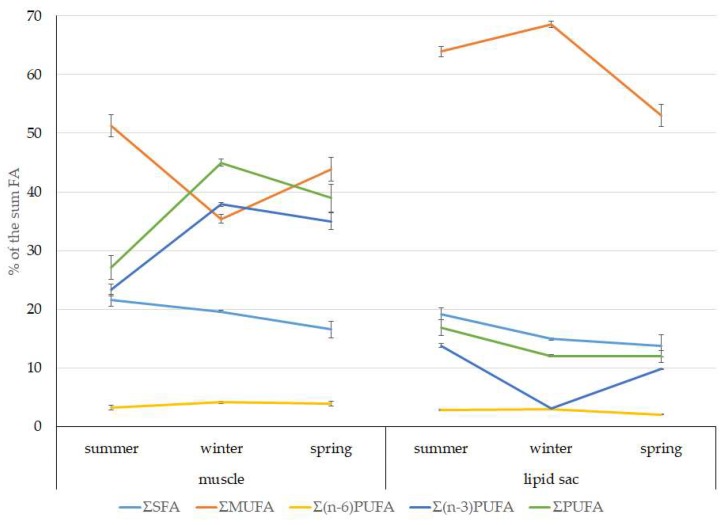
Seasonal dynamic of the saturated FA (SFA), monounsaturated FA (MUFA), polyunsaturated FA (PUFA) levels, including (n-3)PUFA and (n-6)PUFA (% of the sum of FAs) in muscle and the lipid sac in postlarval (L3 developmental stage) daubed shanny from Kongsfjord. The data presented as M ± SE.

**Figure 7:**
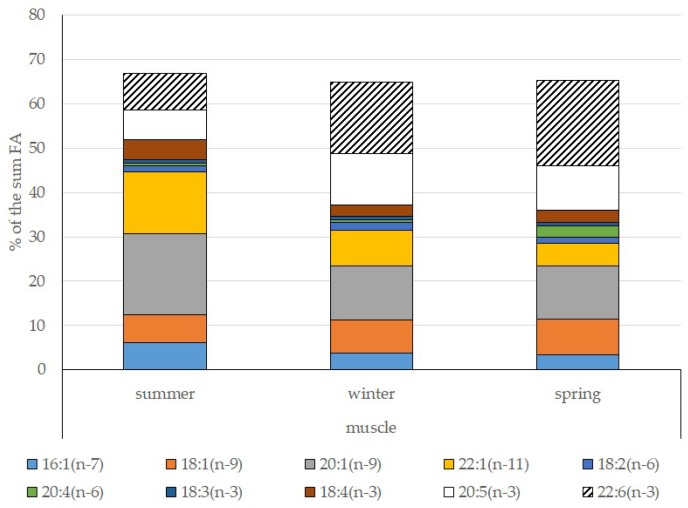
Seasonal dynamic of certain MUFA and PUFA levels (% of the sum of FAs) in muscle in postlarval (L3 developmental stage) daubed shanny from Kongsfjord.

**Figure 8:**
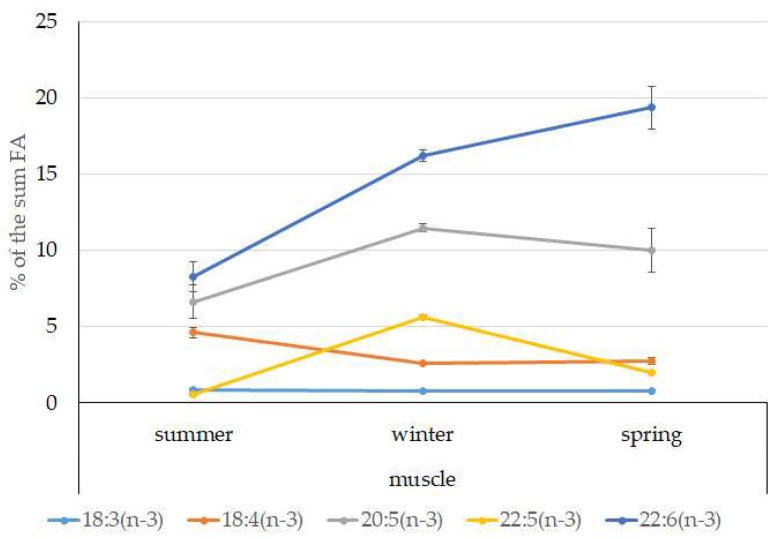
Seasonal dynamic of certain (n-3)PUFA levels (% of the sum of FAs) in muscle in postlarval (L3 developmental stage) daubed shanny from Kongsfjord. The data presented as M ± SE.

**Figure 9:**
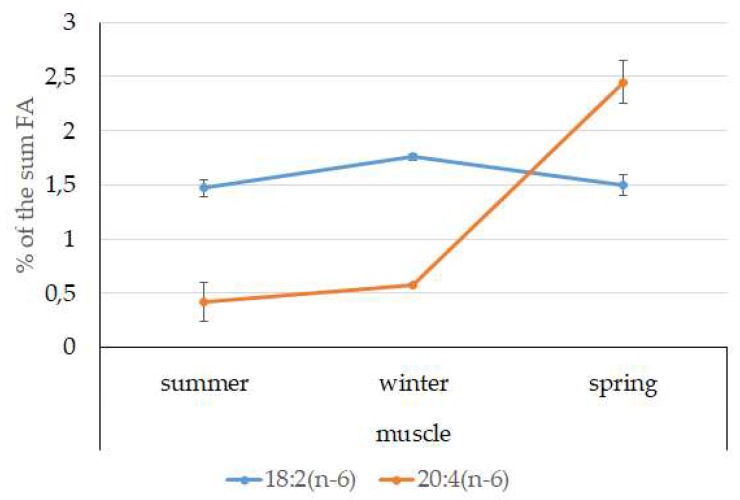
Seasonal dynamic of certain (n-6)PUFA levels (% of the sum of FAs) in muscle in postlarval (L3 developmental stage) daubed shanny from Kongsfjord. The data presented as M ± SE.

**Figure 10:**
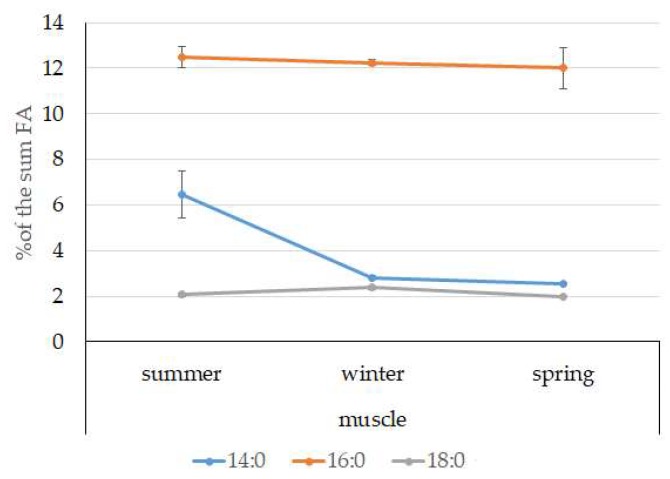
Seasonal dynamic of certain SFA levels (% of the sum of FAs) in muscle in postlarval (L3 developmental stage) daubed shanny from Kongsfjord. The data presented as M ± SE.

Among FAs in the muscles of the studied fish in summer, monounsaturated FAs (MUFAs) were dominant (51.3% of the FA sum) due to 20:1(n-9) and 22:1(n-11) FAs (18.3% and 13.9% of the FA sum, respectively), which pointed to the prevalence of *Calanus* copepods in the diet (Figure 6 and Figure 7). The muscles were also supplied with 16:1(n-7) and 18:1(n-9) FAs (6.2% and 6.2% of the FA sum, respectively), which are of phytoplanktonic origin (Figure 7). The second position in the FA profile in the muscles belonged to PUFAs (27.1% of the sum of FA), due to the (n-3)PUFA (23.3% of the sum of FAs) (Figure 6), including the essential 20:5 (n-3) and 22:6 (n-3), as well as another FA 18:4(n-3) (6.6%, 8.3%, and 4.6% of the FA sum, respectively) (Figure 8). The contribution of the (n-6) PUFA was not high, with 3.3% of the sum of FAs (Figure 6), where 18:2(n-6) and 20:4(n-6) accounted for 1.5% and 0.4% of the sum of the FAs, respectively (Figure 9). The saturated fatty acid (SFA) level was 21.6% of the sum of FAs due to 16:0, 14:0, and 18:0 FAs (12.5%, 6.5%, and 2.1% of the sum of FAs, respectively) (Figure 6 and Figure 10).

The lipid profile of muscles in winter had a different quantitative FA composition with the dominance of PUFAs, in which the level increased, compared to summer, to 45% of the sum of FAs due to (n-3)PUFA (37.9% of the sum of FAs), among which the essential 20:5(n-3), 22:6(n-3), and 22:5(n-3) FAs increased in proportion (11.5%, 16.2%, and 5.6% of the sum of FAs, respectively) (Figure 8). The level of (n-6)PUFA had also increased since summer, to 4.2% of the sum of FAs due to increased contents of 18:2(n-6) and 20:4(n-6) FAs (1.8% and 0.6% of the sum of FAs, respectively). MUFA amounts in the muscles of *L. maculatus* in winter decreased to 35.4% of the sum of FAs, mainly at the expense of 20:1(n-9), 22:1(n-11) FAs (12.2% and 8.1% of the sum of FAs, respectively). The decline in 16:1(n-7) and 18:1(n-9) FAs in fish muscles in winter was the least pronounced, but reliable compared to summer levels (3.8% and 7.5% vs. 6.2% and 2.1% of the sum of FAs, respectively).

MUFAs dominated in muscles in daubed shanny in spring, but their amounts were reliably lower than in summer (31.6% vs. 51.3% of the sum of FAs). The springtime FA profile of muscles featured a PUFA level that was significantly lower than that in summer or winter at the expense of the essential 20:5(n-3) and 22:6(n-3) FAs, with 11.2% and 19.3% of the sum of FAs, respectively. Curiously, it was in spring that the quantities of another essential FA of the (n-6)PUFA (arachidonic FA) reached a maximum in *L. maculatus*, at 2.5% of the sum of FAs. Variations in PUFAs and their individual FAs were correlated with changes in the content of some minor PL classes, namely, PI and PS.

**Figure 11:**
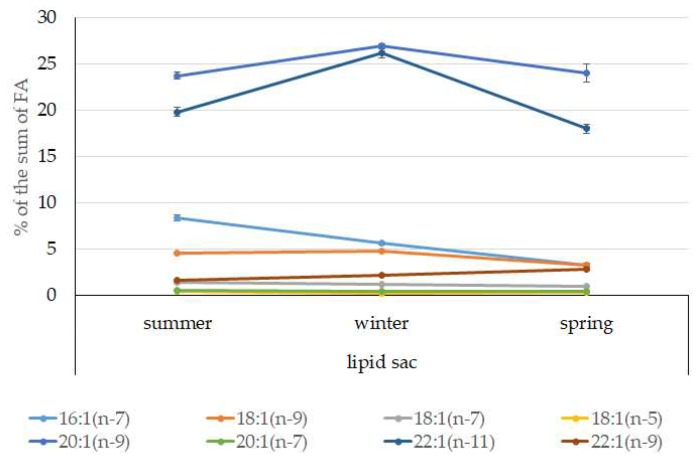
Seasonal dynamic of certain MUFA levels (% of the sum of FAs) in the lipid sac in postlarval (L3 developmental stage) daubed shanny from Kongsfjord. The data presented as M ± SE.

**Figure 12:**
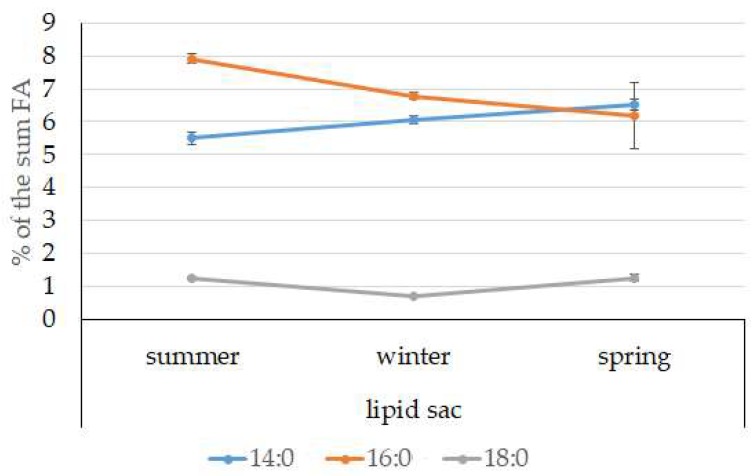
Seasonal dynamic of certain SFA levels (% of the sum of FAs) in the lipid sac in postlarval (L3 developmental stage) daubed shanny from Kongsfjord. The data presented as M ± SE.

**Figure 13:**
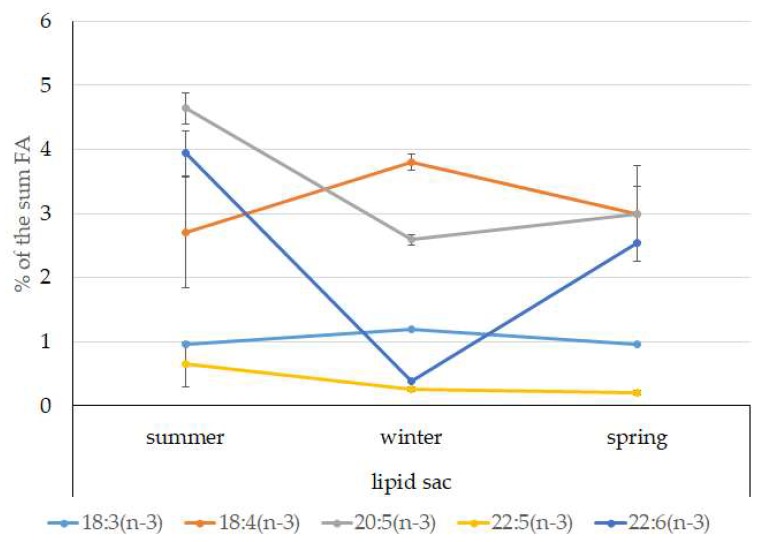
Seasonal dynamic of certain (n-3)PUFA levels (% of the sum of FAs) in the lipid sac in postlarval (L3 developmental stage) daubed shanny from Kongsfjord. The data presented as M ± SE.

The dominant group in the lipid sac (all seasons) was MUFAs (Figure 11), mainly due to 20:1(n-9) and 22:1(n-11) FAs (respectively 63.9%, 23.7%, and 19.8% of the sum of FAs (Figure 8)), derived from a copepod diet of *Calanus* spp. *Calanus* copepods synthesize these FAs de novo and are the main food for postlarvae at the L3 stage. Another MUFA present was 16:1(n-7), amounting to 8.4% of the sum of FAs. It is produced by diatoms and consumed by postlarvae through food. The second largest FA class in the lipid sac of the daubed shanny was SFAs, due to 16:0, 14:0, and 20:0 (19.2%, 7.9%, 5.5%, and 4.1% of the sum of FAs, respectively), which are the main constituents of the biomembranes of the lipid sac (Figure 12). Another component of membrane structures, alongside SFAs, are PUFAs (16.9%), due to (n-3)PUFA 20:5(n-3) and 22:6(n-3) FAs, which contributed 13.8%, 4.6%, and 3.9% of the sum of FAs, respectively (Figure 13). The FA profile of the lipid sac of postlarvae in winter was noted for its elevated MUFA content (68.6% of the sum of FAs), due to the dietary 20:1(n-9) and 22:1(n-11) FAs.

**Figure 14:**
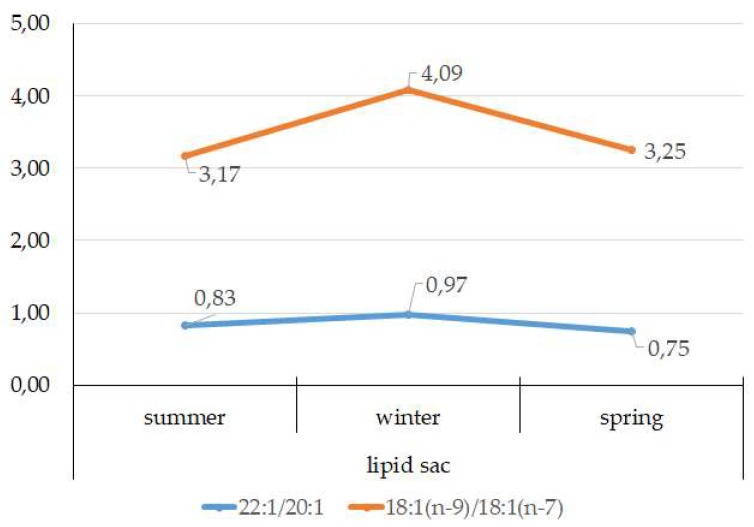
Seasonal dynamic of 22:1/20:1 and 18:1(n-9)/18:1(n-7) ratios in the lipid sac in postlarval (L3 developmental stage) daubed shanny from Kongsfjord. The data presented as M±SE.

According to the 18:1(n-9)/18:1(n-7) ratio, juveniles belong to the group of zooplanktonophages. This is also indicated by the dominance of 20:1(n-9) and 22:1(n-11) MUFAs, which are copepod biomarkers. Seasonal variations in the ratio of these FAs allow for tracking of seasonal variations in the species composition of copepods, the main food objects of juveniles, and their availability in different seasons of the year (Figure 14).

## 4. Discussion

### 4.1. Histomorphology of the Lipid Sac of Postlarval L. maculatus

The lipid sac (LS) is found only in the circumpolar fish *L. maculatus* of the Stichaeidae family. The LS is a unique temporary structure and acts as one of the mechanisms for physiological and biochemical adaptation, helping young fish to survive and develop in northern latitudes [27,28,29,31,32,33].

Interestingly, the LSL and the YSL of bony fish have several important similarities, although these structures are not analogous or homologous: during development, their nuclei grow and their shape becomes more complex with different heterochromatin content throughout, and the functioning of symplasts intensifies during transitory developmental periods. A symplast with polyploid nuclei is one of the most widespread organizational variants of the extraembryonic system.

### 4.2. Dynamics of Total Lipid and Certain Lipid Classes in the Lipid Sac and Muscles of Postlarval L. maculatus

The lipid sac serves as a lipid depot, securing the viability and resilience of pelagic postlarvae in the severe environment of Svalbard waters, including prolonged winter and conditions of polar night. In addition, we found a significant decrease in TL content in the lipid sac by spring.

Pelagic postlarvae accumulate lipids during summer, which is the most productive but the shortest period in the Arctic. Typically, pelagic juveniles of *L. maculatus* at the L3 developmental stage are zooplanktivourous, fattening in the summer by consuming high energy food such as copepods, most abundantly represented by *Calanus finmarchicus* and *Canalus glacialis*. Copepods are the principal food components for the daubed shanny at this stage of development [29,32].

It has been presented [47,48] that copepods account for over 60% of the total number of species in the zooplankton community in Svalbard waters, the dominant ones being boreal oceanic *C. finmarchicus*; Arctic neritic *C. glacialis*; arctoboreal *Pseudocalanus* sp.; cosmopolitan *Oithona similis*; Arctic bathypelagic *Metridia longa*; oceanic cold-water *Microcalanus* sp.; and arctoboreal neritic *Acartia longiremis*. *C. finmarchicus* was shown to be abundantly present in Kongsfjord [47] due to the specific hydrobiological characteristics of this fjord. It appears that, like some other Arctic aquatic organisms (e.g., zooplankton) that accumulate lipids in the body or in its special formations, deposition of a certain amount of lipids is a prerequisite for overwinter survival, as well as for providing energy for the growth and development of larvae and postlarvae of *L. maculatus*, as well as processes of metamorphoses. Furthermore, lipid accumulation in the lipid sac improves the buoyancy of postlarvae, and enables them to stay in the high-productive pelagic zone.

TLs in the muscles of young *L. maculatus* are accumulated and utilized exclusively for their direct function. The highest range of storage-lipid variation in the muscles of postlarval *L. maculatus* proves that these lipids are utilized to maintain energy-intensive metabolic processes that provide relevant postlarval locomotion. The decrease of storage lipids in TAGs that form from summer to winter points to their preferred utilization by the organism, as well as to a reduction of their amount due to limited nutrition in winter. The increase of TAGs and minor classes of storage lipids—represented by Chol esters and wax esters—in the muscles of the postlarval daubed shanny from winter towards spring can be regarded as an indicator of active feeding resumption, mainly on zooplankton. In muscles, the content of Chol, which is a major structural component of biomembranes, did not vary among seasons.

The resistance of aquatic organisms’ biomembranes to various environmental factors largely depends on their lipid components. Changes in PL content and the ratios of their individual classes (mainly in muscles and the lipid sac) are key compensatory mechanisms in organisms that secure optimal performance of a large number of membrane-bound enzymes and their complexes [49] under a range of temperature conditions in the studied seasons. Furthermore, variations in PL quantities (mainly in PE, PS, and PI) and in FA components maintain and ensure required membrane fluidity, which is a property associated with adaptation to variable temperature conditions in the muscles of the daubed shanny [50,51,52]. Seasonal variations of water temperature in Kongsfjord (4.2 °C in summer, 1 °C in winter, and 0.5 °C in spring) activate the adaptive modifications of lipid components and influence the physical and chemical properties of cellular and subcellular membranes, as indicated by the PC to PE ratio in the muscles of postlarvae, as previously demonstrated for other aquatic organisms [53,54,55]. The mechanism of biomembrane adaptation to temperature reduction demonstrated for the muscles and gills of dark flounder *Pseudopleuronectes obscurus* [54] is based on oppositely directed variations of FAs within PC and PE: a rise in saturated FAs to polyunsaturated FA (SFA/PUFA) molecular form and a reduction in the ratio MUFA/PUFA, and PUFA/SFA in PC with the opposite trend observed for PE. Such changes in a certain set of the molecular forms of major membrane PLs are meant to maintain functional activity of the inner lipid monolayer and do not affect transformations in the outer monolayer of biomembranes, i.e., FA components of PLs are redistributed without a change in PL composition.

Functional biomembrane activity in the lipid sac, similar to the muscles of *L. maculatus* postlarvae exposed to variable temperature and foraging conditions in the studied seasons, is maintained both by changes in the quantities and ratios of individual PL classes, as well as through other biochemical mechanisms. One is a change in the Chol/PL ratio, which indicates variations in the microviscosity of biomembranes and their permeability mediated by key membrane lipids. A lower Chol/PL ratio in summer points to a higher permeability of biomembranes in the lipid sac as a result of utilization of lipid components for maintaining metabolic activity in this period. Thus, the lipid sac is a unique, metabolically active organ exemplifying successful physiological and biochemical adaptation of the young daubed shanny to living in high-latitude Arctic waters. Lipid sac formation, which begins with exogenous feeding in the larvae, is considered as an example of physiological and biochemical adaptation to fish habitats in the pelagic zone, contributing to their successful growth [29,56].

### 4.3. Seasonal Dynamic of Certain Fatty Acids of Total Lipids in Postlarval L. maculatus

The qualitative and quantitative composition of FA components of TLs in the muscles and lipid sac of *L. maculatus* postlarvae inhabiting Kongsfjord in summer reflects the dietary regime and specialization in this period, as well as tissue- and organ-specificity, which is defined both by metabolic features as well as by physiological and biochemical functions performed by these organs under given environmental conditions. A manifestation of the latter is the selective integration of FAs derived from food into the lipid structure of muscles and the lipid sac, thus providing the fish with energy and structural components that the organism needs. This adaptive modification satisfies the organism’s physiological demand for the accumulation of lipids with a certain structure in order to maintain a relevant metabolic level during long winters. Thus, the inclusion of 20:1(n-9) and 22:1(n-11) into the structure of energetic lipids (mainly TAGs) renders them metabolically active owing to the physical and chemical properties of these FAs, and helps satisfy the energy demand of daubed shanny postlarvae, even when water temperatures are low. Stage CV copepodites and adult copepods of the *Calanus* species have quite a high share of 20:1 and 22:1 FAs within the dominant energetic lipids class, namely, wax esters.

It was previously demonstrated that phytoplanktonic PUFAs can be partially transformed by zooplankton into SFAs and MUFAs, and can be deposited in their body as waxes, a long-term energy depot [57]. Thus, postlarval *L. maculatus* obtains essential phytoplankton-derived 20:5(n-3) and 22:6(n-3) FAs while feeding on zooplankton, which is a link for the transfer of essential FAs from primary producers to zooplankton feeders and higher-order consumers in high-latitude marine ecosystems, especially during the most productive summer period, when fish actively forage to accumulate lipid reserves.

Previously [29,58], it was shown that the dominance of young individuals (L2 and L3 developmental stages) in winter in Kongsfjord and older-age individuals (L5 stage) in the near-bottom layers of the water column corresponds with the division of the young daubed shanny into pelagic and demersal ecological groups. Similar changes in lifestyle and living conditions are also found in Antarctic fish [59,60]. In addition, studies [29,57] of the growth and early development patterns of the daubed shanny showed an increase in the length and weight of juveniles with age, increased growth at the age of 2+ (L2 stage), which is connected with the transition from phytoplankton feeding to high-energy zooplankton, while energy in the form of lipids is used on active growth. Thus, nutrition basis at the L1 development stage was phytoplankton-dominated by dinophyte algae, while a high level of biomarker 20:1(n-9) and 22:1(n-11) FAs from stage L2 reflects the nutrition of high-energy zooplankton of the *Calanus* genus, which is able to synthesize these FAs de novo [29,61]. Thus, the unique role of the postlarval daubed shanny in the transfer of matter and energy in the Arctic pelagic food chain with the participation of key chain links (phyto- and zooplankton), even in winter conditions, was also determined in this study. Interestingly, these results support the hypothesis about peculiarities of marine ecosystems functioning as a whole during polar nights in terms of the fact that the interaction of ecosystem components is determined by the season, e.g., despite the specific photoperiod, they actively interact with each other in winter, and zooplankton continue to make up a certain share in pelagic community structure, providing food to pelagic fish. The change of food objects and, accordingly, the type of food from phyto- to zooplankton in the juvenile daubed shanny, and then to carnivorous in adult individuals, was also shown for Antarctic silverfish *Pleurogramma antarcticum* [62], which is most numerous in East Antarctic waters [63].

By accumulating PUFAs in the TLs of its muscles, the postlarval daubed shanny ensures that membrane-bound enzymes and their complexes can function optimally when the functional load on the organ (e.g., swimming function and rheotaxis) or environmental conditions (e.g., temperature and salinity) changes. Polyunsaturated FAs largely define the inner structure of biological membranes and conditions for the activity of integral membrane proteins [64]. Some previously reported experiment data [64,65] suggested that if lipids with polyunsaturated chains are involved in the formation of the specific microenvironment of integral proteins then, by virtue of their physical and chemical properties, they can contribute to maintaining the relevant conformational mobility of these proteins and mitigate the negative effects of temperature changes on their activity, thus helping them to function normally. Furthermore, a sufficient supply of PUFAs during the development of *L. maculatus* postlarvae is necessary for their normal growth and development, since these compounds play important roles in regulating the activity of nerve cells in the formation of the vision system in fish, which is particularly crucial for the pelagic young. Young fish suffering a deficit of PUFAs may exhibit abnormal behavioral reactions.

The elevated PUFA level in the muscles of postlarval *L. maculatus* in winter is most probably due to their accumulation during the fattening season, which is a necessary precondition for the optimal functioning of integral membrane enzymes under low temperatures (the temperature in Kongsfjord in winter is 1 °C) and for these PUFAs to co-maintain their general membrane structure together with the basic MUFAs in the PL structure, 18:1(n-9) FAs, whose level remains constant in the winter season. With regard to temperature adaptations, one should always keep in mind the important role of biomembranes and their sensitivity to changes in ambient temperature. Many processes going on in cellular and subcellular membranes are essential for the organism, including the biosynthesis of the membrane itself. It is membranes that set the temperature limits within which all systems can function normally and an organism remains viable.

An elevated content of the essential 20:4(n-6) FA in the muscles of postlarvae may point to the ongoing synthesis of lipid signal mediators, which are synthesized in a majority of tissues and are involved in the regulation of a series of physiological processes: immune response, inflammatory reactions, and functioning of vision and the nervous system [66,67].

The FA profile of the lipid sac of the postlarvae in winter was noted for its elevated MUFA content (68.6% of the sum of FAs) due to dietary 20:1(n-9) and 22:1(n-11) FAs, corroborating its key role as an energy storage organ and demonstrating a successful accumulation of lipids, both in quality and quantity, during the fattening season. Studies showed [47] that the wintering stocks of *C. glacialis* are chiefly made up of stage IV–V copepodites, concentrated at 150 m depth, and for *C. finmarchicus,* this is copepodite stage V, living at 50–150 m depths. An 18:1(n-9)/18:1(n-7) ratio allowed us to categorize the daubed shanny as a carnivorous group of animals. Juveniles belong to the group of zooplanktonophages according to the 18:1(n-9)/18:1(n-7) ratio (above 1).

The lipid sac of the postlarvae contained lower levels of PUFAs in winter, with a simultaneous rise in Chol content compared to that in summer. This combination may point to an increase in biomembrane viscosity and a corresponding reduction in the activity of lipid transport from the lipid sac to preserve them for utilization later during the winter.

There probably exists some threshold metabolic level in this age group (L3 developmental stage) of *L. maculatus*, tightly linked to the stressfulness of environmental conditions in winter, and until this threshold is reached, lipids stored in the lipid sac are moderately utilized to support basic functions. This statement is confirmed by the reduced MUFA level (to 53% of the sum of FAs), including 20:1(n-9) and 22:1(n-11), in the lipid sac of postlarvae. However, the spring sampling of the young daubed shanny in Kongsfjord took place on 21 April, when phytoplankton was blooming (*Chl a* level was within 1.6–1.9 µg/L; 51% due to diatoms), and *Calanus* spp. copepods were present in the area [68]. Phytophagous copepods with pronounced ontogenetic (seasonal) migrations display a distinct correlation with the onset of the high-productivity period, when the vernal phytoplankton outburst takes place. At this point, the overwintered stage V–VI copepodites concentrate close to the surface for the start of the breeding season [47]. Meteorological conditions in this period of spring strongly fluctuated (wind speed up to 8.2 m/s and its directions), causing the *Chl a* level to fall below 0.5 µg/L and resulting in vertical water mixing and cooling, especially down to a depth of 60 m. The vertical redistribution of the phytoplankton biomass happening in the process caused transient breaks in its blooming, and a redistribution of phytoplankton species ratios and its low biomass were therefore observed [69,70]. Following these events, in the period from 28 April to 12 May, the conditions at our study site in the fjord were shown to have stabilized [68]. Thus, the lowest MUFA level in the lipid sac was detected in springtime, possibly due to their utilization within lipids to support the vitality of postlarval fish under the specific conditions of this period.

Observed quantitative MUFA and PUFA variations testified to their particular roles and evidence that the FA composition of lipids is specific to the studied organs, in accordance with the function they perform under given and variable environmental conditions. Certain FAs are retained in the structure of lipids to enhance their metabolic value, both to facilitate a general adaptation of postlarvae to overwintering, and to provide specific compensatory reactions adjusting an organism to seasonal environment.

MUFAs dominated in muscles in the studied fish in spring, but their amounts were reliably lower than those in summer (31.56% vs. 51.30% of the sum of FAs). The springtime FA profile of the muscles of the postlarvae featured a PUFA level that was reliably lower than that in summer or winter at the expense of the essential 20:5 (n-3) and 22:6 (n-3) FAs—11.15% and 19.34% of the sum of FAs, respectively. Curiously, the quantities of another essential FA of the n-6 family (arachidonic FA) reached a maximum in *L. maculatus* (2.45% of the sum of FAs) in spring. Variations in PUFAs and their individual FAs were correlated with changes in the content of some minor PL classes, namely, PI and PS. This resulted in an alteration in the activity of biomembrane enzymes due to modification of their lipid environment. Furthermore, in a stressful environment, FA components perform the function of lipid mediators, becoming integrated in the structure of their molecules. As mentioned above, the water temperature in Kongsfjord is 0.5 °C in spring. The observed lipid and FA profile of postlarval muscles in springtime is indicative of their role in the adaptation to the highly variable meteorological and hydrological conditions in the habitat in this season, which most probably demand intensified locomotion, orientation in the water column, and relevant provision for corresponding metabolic functions (energy supply and signal transduction in tissue by means of lipid-type mediators). In addition to their structural function, the minor PI and its physiologically active metabolites (inositol, triphosphate, diacylglycerols) are known to modulate the activity of the phosphotransferase protein kinase C, which is particularly important for cell growth and differentiation in young fish [71].

Thus, compensatory modifications in the composition of lipids and their FA components in the muscles and lipid sac of typically pelagic L3 stage postlarvae of the daubed shanny living under highly variable environment (primarily temperature and foraging conditions, as well as photoperiod) secure the viability of this Arctic fish species. Owing to some of its specific physiological and biochemical traits (e.g., the lipid sac in its postlarvae), the daubed shanny has a marked ecological role and significance in the marine ecosystem’s food chains [72].

## 5. Conclusions

This paper presents results on the seasonal dynamics of lipids and their FA components in the muscles and the lipid sac of postlarval *L. maculatus* inhabiting Kongsfjord in the summer, winter, and spring in the Arctic, with particular attention paid to the role of lipids in the adaptation of young fish to seasonal variations in temperature and photoperiods (abiotic factors), as well as an important biotic factors associated with the number, species composition, and availability of food objects. Biochemical analysis was carried out for the typically pelagic L3 developmental stage of the juvenile *L. maculatus*, which is predominantly zooplanktovorous.

Both general and numerous specific biochemical adaptation mechanisms of the compensatory and exploitative variety, which were found in daubed shanny juveniles, involve lipid participation, and their high plasticity can be explained by the evolutionary “youth” of this species, formed due to the movement of its Pacific ancestors to the North Atlantic about 3.0–3.5 million years ago [12], in addition to prolonged spatial continental isolation, which led to formation of an endemic species of the *Leptoclinus* genus [12].

Among specific adaptations, we have to highlight:

- The presence of a unique formation, in the form of the *lipid sac*, is a specific ecological and biochemical adaptation in the early development of daubed shanny. The lipid sac symplast of the juvenile daubed shanny is a structure that was discovered and described for the first time;
- The lipid sac accumulates a large amount of lipids, mainly TAGs and specific MUFAs of food origin, 20:1(n-11) and 22:1(n-9), indicating that *Calanus* spp., which form the basis of the Arctic zooplankton biomass, dominate in the diet of juveniles. The established seasonal dynamics and changes in the ratios of these FAs indicate different species composition and accessibility of copepods in the studied fjords (mainly Kongsfjord, Svalbard) for the pelagic juveniles of the daubed shanny. Tracking the movement of food-derived FAs determines the basis for the chemoindication of qualitative and quantitative relationships between zooplankton and zooplanktophages in Arctic marine food chains;
- A slight but significant increase in the level of derived from food FAs in the lipid sac in spring is synchronized with the beginning of the spring bloom of phytoplankton and the appearance of stage V–VI copepods in the pelagic water layers to start a reproductive period. Interestingly, the inclusion of long-chain 20:1(n-9) and 22:1(n-11) FAs in the structure of energy lipids (mainly TAGs) of the lipid sac of the daubed shanny maintains their energy value, which brings on the corresponding needs of juveniles, even at low temperatures during the long winter period in the Arctic;
- A minor class of energy lipids in the form of Chol esters and waxes is only found in the lipid sac of juveniles in the winter–spring period, which reflects the peculiarities of their nutrition and lipid accumulation.

Among the general adaptation mechanisms, a high unsaturation of lipids was shown for daubed shanny juveniles due to their FA components, characteristic of inhabitants of marine ecosystems of northern latitudes. A high degree of lipid unsaturation is supported, first, due to the dominance of MUFAs, among which oleic acid 18:1(n-9), that is present in the structure of almost all lipids, both structural and primarily storage lipids, is one of the main biochemical component. The second position is often competitively occupied by PUFAs and/or SFAs. In this case, we should mention the relationship between the results of this work and theoretical studies of the properties of FA chains (the shape and flexibility of FA chains) in an unperturbed state, obtained by computer simulation with the Monte Carlo method [63,64,73,74]. In particular, it was found that FA chains (16:0, 18:1(n-9)*cis*, 18:2(n-6)*cis*, 18:3(n-3)*cis*, 20:3(n-6)*cis*, 20:4(n-6) *cis*, 20:5(n-3)*cis*, 22:5(n-3)*cis*, 22:6(n-3)*cis*), i.e., key FAs for the studied fish, have equal longitudinal sizes due to which molecular substitutions, manipulations, and complementarity (in the properties) of these FAs in the PL structure achieve constant thickness of the membrane hydrocarbon layer and adjust its fluidity, which ensures the functionality of the lipid bilayer under changing environmental factors.

In addition, for fish of the Stichaeidae family, both juveniles and adults [75], lipid unsaturation is achieved due to PUFAs, especially EPA and DHA, in muscle tissue, which is consistent with the increased motor activity of fish in specific habitat conditions (currents, turbulent flows, etc.) and, ultimately, is directed to ensure homeostasis of the internal environment to maintain optimal activity of membrane-bound enzymes with a general increase in the level of metabolism. At the same time, attention should be drawn to the fact that this phenomenon is apparently based on a mechanism that is associated with certain properties: increased flexibility (which is associated with the fluidity of the biomembrane) for PUFAs, i.e., EPA and DHA, compared to SFAs. For example, with an increase in the metabolic load in fish muscle tissue in a specific environment (low temperatures, increased concentration of dissolved oxygen, fluctuations in salinity, etc.), the full functioning of biomembranes is maintained due to the control of such signs as thickness and resistance to mechanical action (with the active work of proteins) and the provision of a thermally insulating function for membrane-bound enzymes in the individual domains of the lipid bilayer. For young marine fish living at low temperatures, the increased level of EPA and DHA is due to their adaptive significance of overcoming the negative impact of fluctuations of this factor on the growth and development of juveniles. Thus, the experimental results of FA status in fish of the northern seas obtained in this work are consistent with the previously formulated concept of the functions of polyunsaturated chains [63,64,73,76].

The conducted studies allowed us to identify the general and specific features of the biology of the daubed shanny, an ecologically important representative of the Arctic ichthyofauna. Further research on the daubed shanny as a component of the Arctic trophic net will be focused on the study of the abilities and/or limitations of biosynthesis of certain MUFAs and PUFAs, including food-derived ones, that can compensate environmental changes and maintain the presence of the species in the northern marine ecosystems.

## Figures and Tables

**Table 1:** Hydrological characteristics of the sampling sites of *L. maculatus* postlarvae in Kongsfjord.

Sampling Fjord	Kongsfjord		
Season/Parameter	T (°C)	Salinity (%)	Depth (M)
Summer (end of July-beginning of August)	4.2	34.7	100
Winter (mid. January)	1	34.8	125
Spring (mid. April)	0.5	34.95	100

**Table 2:** Seasonal dynamics of total lipids and their individual classes (% of dry weight; % of total phospholipids for individual phospholipid classes) in the muscle and lipid sac of postlarval daubed shanny from Kongsfjord.

Season	Summer		Winter *		Spring	
Tissue/Organ	Muscle	Lipid Sac	Muscle	Lipid Sac	Muscle	Lipid Sac
n	15	15	40	40	12	12
TL	27.9 ± 3.00^b^	64.4 ± 2.1^a^	13.9 ± 0.5^a^	75.9 ± 2.8^b^	13.4 ± 0.4^a^	46.9 ± 2.9^c^
TAG	12.7 ± 1.8^a^	58.3 ± 3.1^a^	2.4 ± 0.2^b^	68.8 ± 2.5^b^	6.1 ± 1.0^c^	28.1 ± 1.3^c^
Chol esters+wax esters	1.7 ± 0.4^a^	0	0.9 ± 0.1^b^	3.02. ± 0.4	1.7 ± 0.2^c^	2.6 ± 1.0
PL	9.6 ± 1.4^a^	6.1 ± 1.7^a^	7.2 ± 0.3^b^	0.8 ± 0.3^b^	2.5 ± 0.3^c^	6.3 ± 1.3^c^
PI	2.3 ± 1.0^a^	1.1 ± 0.1^a^	3.8 ± 0.2^a^	2.4 ± 0.6^b^	3.0 ± 0.2^a^	2.3 ± 0.5^b^
PS	1.0 ± 0.1^a^	2.0 ± 0.1^a^	3.4 ± 0.2^b^	3.3 ± 0.2^b^	3.1 ± 0.2^b^	1.2 ± 0.4^c^
PE	20.0 ± 1.8^a^	18.1 ± 1.3^a^	26.7 ± 0.8^b^	22.2 ± 1.0^b^	24.6 ± 1.0^c^	20.1 ± 1.4^c^
PC	74.5 ± 2.7^a^	75.2 ± 2.1^a^	64.5 ± 1.1^b^	68.2 ± 1.5^b^	62.3 ± 2.0^b^	66.2 ± 2.0^c^
LPC	0.3 ± 0.1^a^	0.8 ± 0.2^a^	0.1 ± 0.0^b^	0.9 ± 0.2^b^	0.2 ± 0.1^b^	1.0 ± 0.8^b^
SM	0.4 ± 0.0^a^	0.5 ± 0.1^a^	1.5 ± 0.1^b^	2.5 ± 0.5^b^	1.0 ± 0.1^c^	2.0 ± 0.2^b^
Chol	3.9 ± 1.2^a^	0^a^	3.5 ± 0.2^a^	3.4 ± 0.4^b^	3.1 ± 0.1^a^	10.0 ± 2.5^c^
Chol/PL	0.4 ± 0.1^a^	0.01^a^	0.5 ± 0.2^b^	4.1 ± 1.2^b^	1.3 ± 0.1^c^	1.6 ± 0.8^c^

TL—total lipid, TAG—triacylglycerol, Chol esters—cholesterol esters, PL—total phospholipids, PI—phosphatidylinositol, PS—phosphatidylserine, PE—phosphatidylethanolamine, PC—phosphatidylcholine, LPC—lysophosphatidylcholine, SM—sphingomyeline, Chol—cholesterol. Different uppercase letters indicate significant (*p* ≤ 0.05) differences when comparing the values in the same type of tissue; n—number of tissues; *—values are given according to [29].
